# Developing novel synbiotic low‐fat yogurt with fucoxylogalacturonan from tragacanth gum: Investigation of quality parameters and *Lactobacillus casei* survival

**DOI:** 10.1002/fsn3.1752

**Published:** 2020-06-29

**Authors:** Maryam Ghaderi‐Ghahfarokhi, Amin Yousefvand, Hassan Ahmadi Gavlighi, Mehdi Zarei, Peyman Farhangnia

**Affiliations:** ^1^ Department of Food Hygiene Faculty of Veterinary Medicine Shahid Chamran University of Ahvaz Ahvaz Iran; ^2^ Department of Food Science and Technology Faculty of Agriculture Tarbiat Modares University Tehran Iran; ^3^ Research and Development Unit Khuzestan Pegah Dairy Company Shush Iran

**Keywords:** *Lactobacillus casei*, low‐fat yogurt, prebiotic, synbiotic, tragacanth gum

## Abstract

Tragacanth gum (TG) displayed a prebiotic activity, but its application was restricted due to high viscosity and deterioration of organoleptic and textural characteristics of food. In this study, TG was depolymerized by Pectinex Ultra Color enzyme followed by membrane separation (30 kDa) to get pectinase hydrolyzed fraction of tragacanth gum (PHFTG) with molecular weight more than 30 kDa. The average molecular weight of PHFTG was 147.7 ± 11.5 g/mol having a fucoxylogalacturonan structure. The prebiotic activity was tested using PHFTG, TG, and inulin as a carbon source. The results showed that the count of *Lactobacillus casei* in PHFTG‐ and inulin‐supplemented media increased significantly during the 48‐hr fermentation (*p* < .05). Five batches of low‐fat set yogurts were prepared by the following formulation: Control (without both *L. casei* and prebiotic), LC‐Cont (containing *L. casei*), LC‐PHFTG (containing *L. casei* + 0.5% PHFTG), LC‐TG (containing *L. casei* + 0.05% TG), and LC‐In (containing *L. casei* + 0.5% inulin), and *L. casei* population and physicochemical properties were monitored during 21‐day storage at 4°C. The number of *L. casei* remained highly acceptable (8.54–8.61 log CFU/g) during 7–21 days of storage in LC‐PHFTG. LC‐In and LC‐PHFTG presented significantly lower syneresis and higher sensory acceptability than LC‐Cont and Control during storage (*p* < .05). LC‐TG displayed weaker body and texture, lower sensory acceptability, and higher syneresis than other samples. This study provides support for expanding the utilization of PHFTG as a potential prebiotic and fat replacer in non‐ or low‐fat dairy products with satisfactory sensory quality.

## Graphical abstract

Tragacanth gum was depolymerized by Pectinex Ultra Color enzyme followed by membrane separation (30 kDa) to get pectinase hydrolyzed fraction of tragacanth gum (PHFTG) with molecular weight more than 30 kDa. The highest *Lactobacillus casei* counts were observed on day 7 for all yogurts. Surprisingly, the counts of *L. casei* were always higher than 8.5 log CFU/g in LC‐PHFTG sample from day 7, and after that, the counts seemed to be stagnant. The *L. casei* count of LC‐In yogurts appeared significantly lower than LC‐PHFTG on day 21 (*p* < .05).

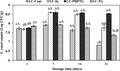

## 1 INTRODUCTION

According to the International Scientific Association for Probiotics and Prebiotics, probiotics are “the live microorganisms, that when administered in adequate amounts, confer a health benefit on the host” (Zendeboodi, Khorshidian, Mortazavian, & Cruz, 2020). Probiotics have a wide array of beneficial effects including anticholesterolemic, antidiabetic, antisclerosis, antiulcerogenic (Roobab et al., 2020), anti‐inflammatory, antiobesity, anticancer, antiangiogenic activities, alleviation of lactose intolerance symptoms, improvement in stomach and colon disorders (Kerry et al., 2018), inhibition of pathogenic bacteria, synthesis of essential micronutrients, and improving the bioavailability of dietary nutrients (Chugh & Kamal‐Eldin, 2020). Nowadays, probiotic‐containing food products are widely accepted by the public and account for 60%–70% of the market share of functional foods (Kareb & Aïder, 2019). For the dairy product to sell with probiotic claims, it should maintain high bacterial viability and survival rate not only at the time of manufacturing and during storage but also while passing through the gastrointestinal tract, which is known to be a harsh environment for probiotics (Kailasapathy & Chin, 2000; Kareb & Aïder, 2019). Accordingly, recent attempts to increase probiotic efficacy include developing synbiotic products through fortification with appropriate prebiotics such as nondigestible substances that selectively stimulate the growth and/or activity of one or limited bacterial species residing in the colon. This approach recuperates host health by modifying the gut microbiota (Stanton, Ross, Fitzgerald, & Van Sinderen, 2005). The consumption of synbiotic dairy products has been associated with some therapeutic functions such as hypolipidemic (Sarfraz et al., 2019), antimicrobial (Shafi et al., 2019), and antihyperglycemic activities (Grom et al., 2020).

On the other hand, due to increased awareness of the harmful effect of excessive intake of dietary fat, consumers may seek for low‐fat dairy products, which may suffer from a lack of sensory quality and body profile available in high‐fat products. Incorporation of prebiotics as a fat replacer to yogurt is one of the conceivable ways to promote health benefits while improving sensory characteristics (Aryana, Plauche, Rao, McGrew, & Shah, 2007; Srisuvor, Chinprahast, Prakitchaiwattana, & Subhimaros, 2013).

Tragacanth gum (TG) is a polysaccharide exudate from the stems and branches of “goat's horn” plant (*Astragalus* sp.), which is often distributed in the mountainous areas of southwest Asia (Kailasapathy & Chin, 2000). TG has been considered as GRAS since 1961 (Generally Recognized as Safe) and could be utilized as a food additive (Anderson & Bridgeman, 1985). TG is a physical mixture of two main fractions: water‐swellable pectic component (*Bassorin*), which can swell and form a gel, and water‐soluble fraction (*Tragacanthin*) as a colloid hydrosol (Balaghi, Mohammadifar, Zargaraan, Gavlighi, & Mohammadi, 2011; Kailasapathy & Chin, 2000). Due to thickening, emulsifying, and stabilizing characteristics, TG offers a wide diversity of applications in foods, pharmaceuticals, textiles, cosmetics, and other industries (Hatami, Nejatian, & Mohammadifar, 2012).

There are many studies on the application of TG in food systems as fat replacer including in nonfat yogurt (Aziznia, Khosrowshahi, Madadlou, & Rahimi, 2008), cheddar cheese (Cooke, Khosrowshahi, & McSweeney, 2013), and low‐fat Iranian white cheese (Rahimi, Khosrowshahi, Madadlou, & Aziznia, 2007), and as a stabilizer in yogurt drink (Azarikia & Abbasi, 2010). Nevertheless, the high viscosity of TG could lead to restriction of applications due to the deterioration of the organoleptic and processing characteristics of food. Also, it may suppress health benefits to some extent (Gavlighi, Meyer, Zaidel, Mohammadifar, & Mikkelsen, 2013). In recent years, increasing attention has been paid to developing processes for enzyme‐catalyzed production of prebiotics from natural polysaccharide source. Such methods, if accompanied by membrane technology (ultra‐ or nanofiltration), could decrease the viscosity of polysaccharides and, at the same time, generate bioactive oligosaccharides with different structural properties and prebiotic potential (Pinelo, Jonsson, & Meyer, 2009). During enzymatic depolymerization of TG, viscosity reduction was attained via molecular weight reduction of the native gum.

Gavlighi, Michalak, Meyer, and Mikkelsen (2013) used enzyme depolymerization and two‐step membrane separation (2 and 10 kDa membranes) to produce three fractions of TG with different molecular weights. Interestingly, TG fractions displayed a noticeable prebiotic activity in single‐culture fermentations of different probiotic strains, which attributed to their *L*‐fucose‐substituted structural moiety. However, to the best of our knowledge, there has been no study on employing enzyme hydrolyzed TG into the food system as a source of prebiotic and fat replacer. The main objectives of the current study were hence to: (a) apply the enzyme polymerization and one‐step membrane separation for the production of pectinase hydrolyzed fraction of tragacanth gum (PHFTG) with molecular weight more than 30 kDa; (b) compare the prebiotic potential of inulin, native TG, and PHFTG on *Lactobacillus casei*; and (c) manufacture the potential synbiotic yogurts by the use of different prebiotics and evaluation of physicochemical properties, organoleptic characteristics, and *L. casei* survival during 21 days of cold storage.

## 2 MATERIALS AND METHODS

### 2.1 Material and bacterial strains

TG exudate from *Astragalus gossypinus* was sourced from a local herbal store (Shahrekord, Iran). The flakes were grounded using a coffee miller, passed through a 0.2‐mm sieve, and finally kept in the sealed plastic bag at −20°C until use. Pectinex Ultra Color, 10,000 PECTU/ml, was obtained from Novozymes A/S. This enzyme preparation is approved as a processing aid for industrial food applications. Prebiotic inulin Orafti HPX with polymerization (DP) ≥23 was supplied by Beneo. De Man, Rogosa, and Sharpe broth (MRSB) and agar were purchased from Ibresco (Zist Kavosh Iranian Company). All other culture media and chemical reagents used in this study were of analytical grade.

*Lactobacillus casei* subsp. *casei* (PTCC 1604, ATCC 39,392) was purchased from Persian Type Culture Collection. Thermophilic yogurt culture, which comprised of *Streptococcus thermophilus* and *L. delbrueckii* subsp. *Bulgaricus* (YoFlex Express 1.0), was supplied by Chr. Hansen Company (Chr. Hansen Inc., Milwaukee, WI).

### 2.2 Enzymatic digestion of TG combined with membrane separation

TG solution (10 g/L) was made by dissolving the appropriate amount of TG powder in Milli‐Q water by gently stirring for 2 hr at room temperature. The resultant solution (pH = 5.5–6) was then heated to 50°C. The enzymatic digestion of TG was carried out by applied Pectinex Ultra Color enzyme (equivalent to the addition of 25,000 PECTU/L) at level of 2.5 ml/L on TG solution at a temperature of 50°C, for 120 min in water bath until the viscosity of the solution had dropped from 1.2 to about 0.5 Pa·s. After enzyme deactivation (100°C for 10 min), enzymatically treated TG solution was centrifuged at 10,000 *g* for 10 min and then continuously filtered for 8 hr by using cross‐flow Hydrosart ultrafiltration unit (Vivaflow^®^200, Sartorius Stedim) equipped with stabilized cellulose base membrane with a 30 kDa molecular weight cutoff. The retentate with molecular weight more than 30 kDa was defined as PHFTG. Finally, the collected retentate (PHFTG) was freeze‐dried, stored frozen at −18°C, and applied in prebiotic activity experiment and in yoghurt formulation (Gavlighi, Michalak, et al., 2013).

### 2.3 Characterization of PHFTG

Monosaccharide composition of PHFTG was determined after acid hydrolysis (4 g/L substrate, 2 M trifluoroacetic acid for 2 hr at 121°C) using high‐performance anion‐exchange chromatography coupled with a pulsed amperometric detector. An Azura system equipped with a P6.1L gradient pump (Knauer), a DECADE Elite electrochemical detector (with a gold working electrode E1 = 0.05 V, 0.5 s; E2 = 0.75 V, 0.13 s; E3 = −0.80 V, 0.12 s), and an injection valve Rheodyne 9725i equipped with a 20 μl injection was used. Separations were carried out using a CarboPac PA20 (3 × 150 mm) analytical column (Dionex Corp.; Gavlighi, Meyer, et al., 2013). The sugars were separated with sufficient resolution by using a two‐eluent system comprised of deionized water (18.2 mΩ at temperature of 25°C) and 500 mM aqueous NaOH. Neutral sugars were eluted isocratically with 2.5 mM NaOH for 20 min. Then, a second isocratic elution was carried out at high concentration of NaOH (500 mM) for 10 min to elute any acidic monosaccharides present. The eluention was performed at flow rate of 0.5 ml/min. Re‐equilibration program of column was run before each sample injection using NaOH (100 mM) for 5 min (Balaghi et al., 2011).

Sample preparation for molecular weight determination was conducted following the method of Gavlighi, Michalak, et al. (2013). A multiangle laser light scattering system (HELEOS; Wyatt Technology Corp) composed of TSK G5000 PW column (7.5 × 600 mm, Toso Biosep), UV detector (Waters, 2,487), and refractive index detector (Waters, 2,414) system (HPSEC‐UV‐MALLS‐RI) was utilized to analyze the molecular weight. An aqueous solution of NaNO3 (0.15 M) and NaN3 (0.02% w/v) at flow rate of 0.4 ml/min was used as mobile phase. Bovine serum albumin was employed to determine the volume delays among the UV, MALLS, and RI detectors. The average molecular weight (*M*_w_), specific volume of gyration (SV_g_), and radius of gyration (*R*_g_) were calculated by ASTRA 5.3 software (Wyatt Technology Corp).

### 2.4 Evaluation of prebiotic activity

The strain was preserved at −80°C in MRS broth containing 25% v/v of glycerol. Before experiments, the *L. casei* was propagated twice, through inoculating 1% v/v of culture in 10 ml MRS broth followed by incubating at 37°C for 24 hr under anaerobic conditions provided by the GasPak system (Anaerocult A, Darmstadt; Zomorodi, Asl, Rohani, & Miraghaei, 2011). The cultures were stored at 4°C and subcultured twice in MRSB immediately before each experiment.

Prebiotic potential of TG and PHFTG was assessed through the growth of *L. casei* compared with inulin as a known prebiotic compound. Before the trials, all prebiotics, including TG, PHFTG, and inulin powder, were sterilized by exposure to UV light (254 nm, 40 w) for 15 min (Rubel, Pérez, Genovese, & Manrique, 2014). To check the sterility of the prebiotics, total viable counts were determined on plate count agar before their use. The experiments were conducted in a glucose‐free MRS broth (GF‐MRSB) culture, which was prepared with the same composition of common MRSB by mixing different ingredients in the laboratory as reported by Moreno‐Vilet et al. (2014). The GF‐MRSB was supplemented with 0.5% and 1% (w/v) of TG, PHFTG, and inulin in individual Erlenmeyer flask. GF‐MRSB and commercial MRSB (with 2% w/v of glucose) were used as negative and positive control, respectively. Different culture media were inoculated with activated *L. casei* at 10^4^ CFU/ml. The accurate inoculation level was verified through plate counting immediately after inoculation. After then, different culture media were divided into 10‐ml glass tubes followed by incubation at 37°C under anaerobic condition. At each sampling time (24 and 48 hr), bacterial colonies were enumerated by plating media culture onto commercial MRS agar followed by anaerobic incubation at 37°C for 48 hr. Utilization of different prebiotics by *L. casei* was also monitored by measuring pH and acidity of the media. Total titratable acidity (TTA) and pH values were measured according to AOAC (2005).

### 2.5 Preparation of low‐fat yogurts

Before the yogurt preparation, preweighted and UV‐sterilized TG and PHFTG powder were gradually added into sterilized water. Then, the mixture was heated at 50°C for 60 min, cooled to ambient temperature, and subsequently kept overnight in the fridge for complete hydration (Azarikia & Abbasi, 2010).

Propagation of *L. casei* was performed according to the method of Srisuvor et al. (2013) with some modifications. Fifty microliters of overnight *L. casei* culture was subcultured into the plastic tube containing 50 ml MRSB. Then, the tubes were incubated at 37°C for 24 hr under anaerobic conditions. The biomass of *L. casei* was harvested by centrifugation at 4,000 *g* for 10 min at 20°C and subjected to wash twice with sterilized standard saline solution. Finally, the collected cells were resuspended in 10 ml of UHT milk and used as a *L. casei* culture for the production of yogurts. Thermophilic yogurt culture was added into 1 L sterilized reconstituted skim milk at room temperature. After mixing, 1 ml of this mixture was transferred to 1 L of milk according to the manufacturer's specifications (Pakseresht, Tehrani, & Razavi, 2017).

**FIGURE 1:**
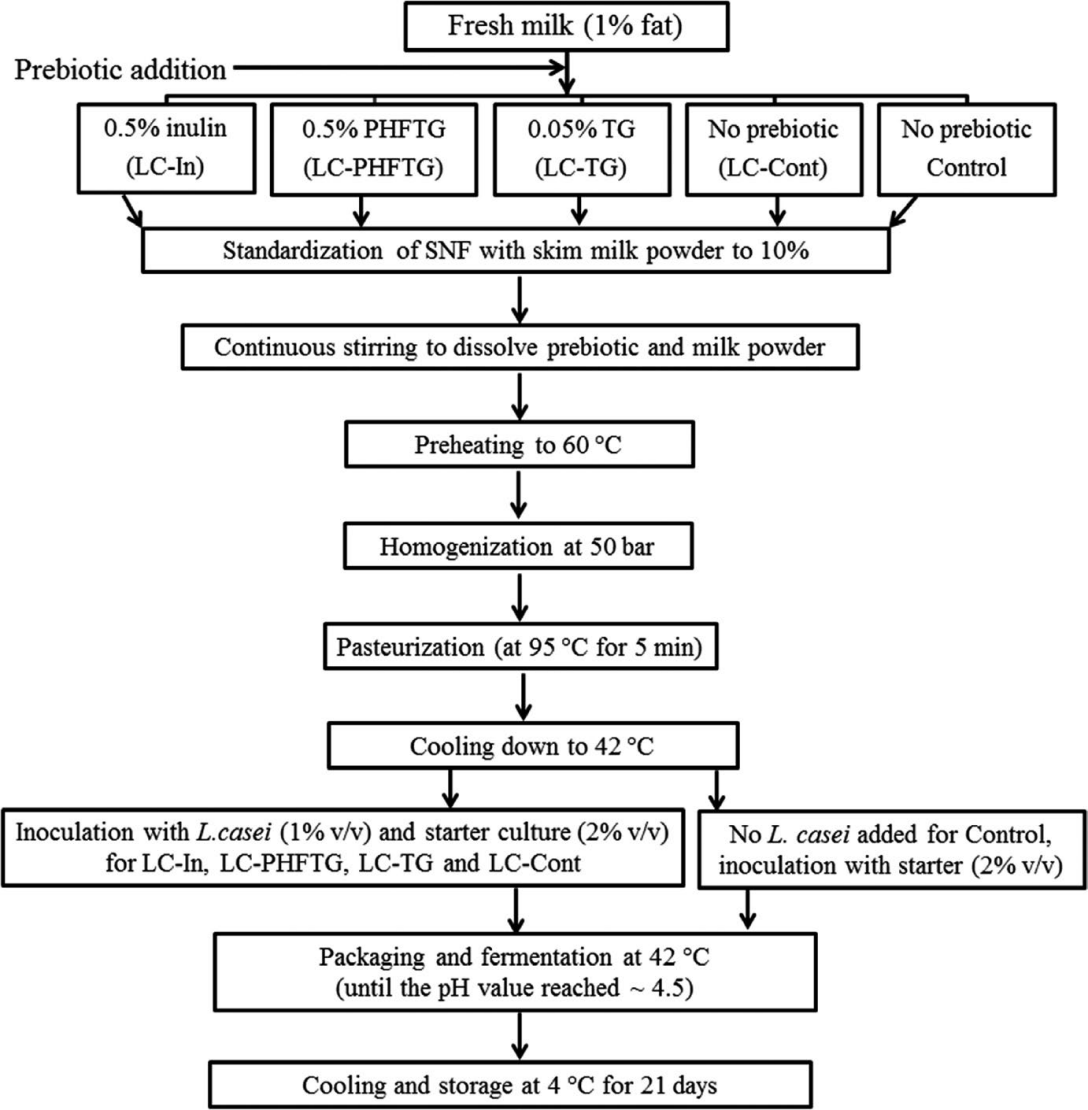
Low‐fat yogurt manufacturing flowchart. Control: yogurt without both *Lactobacillus casei* and prebiotic; LC‐Cont, yogurt containing *L. casei*; LC‐In, yogurt containing *L. casei* and 0.5% inulin; LC‐PHFTG, yogurt containing *L. casei* and 0.5% pectinase hydrolyzed fraction of tragacanth gum; LC‐TG, yogurt containing *L. casei* and 0.05% tragacanth gum; SNF, solid nonfat

Low‐fat set yogurts were manufactured at the Khuzestan PEGAH dairy plant (Iran Dairy Industries Co.). Five yogurt formulations, including Control (without both *L. casei* and prebiotic), LC‐Cont (containing *L. casei*), LC‐PHFTG (containing *L. casei* + 0.5% PHFTG), LC‐TG (containing *L. casei* + 0.05% TG), and LC‐In (containing *L. casei* + 0.5% inulin) were prepared by the procedure depicted in Figure 1. Immediately before incorporation to milk, UV‐sterilized inulin (0.5% w/v) was dissolved in an equal volume of water used for TG and PHFTG hydration. Also, the hydrated TG and PHFTG were gradually added to milk to obtain the final concentrations of 0.05% and 0.5% (w/v), respectively. The milk solid nonfat of all five batches was set to 10% (w/v) with skim milk powder (Mudgil, Barak, & Khatkar, 2016). After production, the yogurts were immediately cooled to 4°C and stored over three weeks at 4°C. The one‐day period displays the measurement of physicochemical properties and *L. casei* count after overnight storage of yogurt samples, and periods 7–21 days represent measurements after 7, 14, and 21 days of storage.

### 2.6 Physicochemical analysis of yogurts

The pH values of the yogurt were recorded using PM12E digital pH meter (Fan Azma Gostar). Also, TTA of yogurt was measured according to the AOAC official method and expressed as % lactic acid (AOAC, 2005).

The extent of syneresis was determined as recommended by Tamime, Barrantes, and Sword (1996). In brief, 25 g of each yogurt batches was weighted on a Whatman paper No. 42 (Whatman), which was placed on the top of a funnel. The method was based on drainage of whey separated from the gel network under the gravity force at 4°C for 2 hr. The percent of syneresis was calculated as the mass of the whey collected in a flask of known weight divided by the initial yogurt mass.

Water‐holding capacity (WHC) was measured based on the centrifugation method reported by Sahan, Yasar, and Hayaloglu (2008). For this purpose, approximately 5 g of yogurt was weighted in the test tube (*M*_i_) and centrifuged at 3556 x g for 30 min at 10°C. The resultant supernatant was discarded, and the expelled precipitate was collected and weighed (*M*_p_). WHC was calculated using the equation:

(1) $$\text{WHC}\;\left(\%\right)=\left[1-\left(M_{p}/M_{i}\right)\right]\times 100,$$

where *M*_i_ and *M*_p_ were the initial weight of the sample and the final weight of the precipitate, respectively.

### 2.7 Enumeration of *L. casei*

The cell population of *L. casei* was counted in freshly produced yogurt samples (after 24 hr of production) and during storage at 4°C as described previously and expressed as log colony‐forming units (CFU) per gram of the product (log CFU/g). The cup of yogurt was agitated with a sterile pipette, and then, 1 g of each sample was transferred into 9 ml of physiological saline solution and homogenized using a vortex mixer for 30 s. An appropriate amount of diluted samples was used for enumeration by the pour‐plate technique. Briefly, the selective media and incubation condition for *L. casei* were MRS–vancomycin agar (pH 6.2; 1 mg/L vancomycin) and anaerobical incubation at 37°C for 72 hr, respectively (Sah, Vasiljevic, McKechnie, & Donkor, 2016).

### 2.8 Sensory evaluation of yogurts

Sensory evaluations were performed on the 10th day of storage using 30 semitrained panelists who were randomly selected from the available students, faculty, and staff members of Shahid Chamran University. Assessors were familiar with the consumption of yogurt and aged between 20 and 55 years with equal distribution of males and females. All yogurts were coded with three digits for identification and served in 100‐ml polyethylene cups at 10–15°C under laboratory conditions, while orders of serving were entirely randomized. Each yogurt sample was scored for appearance, flavor, mouthfeel, body and texture, visual syneresis, and overall preference attributes on a 5‐point hedonic scale anchored on the left with “dislike very much or unacceptable” and on the right with “like very much or acceptable.” Besides, drinking water was provided to the panelists to cleanse their palate before tasting each sample.

### 2.9 Statistical analysis

Analytical data were obtained from analyses of three samples for yogurt trials. For prebiotic activity assay, all experiments were conducted at least twice, and duplicate samples were used for each test. The collected data were submitted to the ANOVA using the General Linear Model procedure. Statistical significance of differences among means was assessed through Tukey's test at a confidence level of *p* < .05. All statistical analyses were performed using Minitab 16 program (Minitab Inc.).

## 3 RESULTS AND DISCUSSION

### 3.1 Monosaccharide composition and molecular weight of PHFTG

The sugar composition of PHFTG is given in Table 1. Among all monosaccharides, galacturonic acid (~35.40%) was the most abundant monosaccharide in PHFTG, followed by xylose (~29.85%) and fucose (~21.88%). Also, arabinose (~8.67%), rhamnose (~2.11)%, and galactose (~1.97%) were present in limited quantities. Only traces of glucose (~0.08%) were detected. The dominance of galacturonic acid, xylose, and fucose in the starting material was previously reported (Gavlighi, Meyer, et al., 2013; Gavlighi, Michalak, et al., 2013). The water‐soluble tragacanthin fraction appears to resemble pectin. It contains linear chains of galacturonic acid and arabino‐galactan structures, and fuco‐xylogalacturonans as the main components, while bassorin is believed to be predominantly built from a mixture of xylo‐ and fuco‐xylo‐substituted polysaccharides (Balaghi et al., 2011; Tischer, Iacomini, & Gorin, 2002). The sugar composition of the fraction with the molecular weight higher than 30 kDa (PHFTG) indicates that pectinase‐assisted degradation has mainly caused releasing the fuco‐xylo‐galacturonan structure. This is in accordance with the attack pattern of the pectinases, which are not capable of attacking the substituted fuco‐xylo‐galacturonic acid structures and seem to preferentially cleave the polygalacturonic acid parts of the TG backbone (Gavlighi, Michalak, et al., 2013). The suspected xylo‐galacturonan, and mainly the fuco‐xylo‐galacturonan stretches were left intact in the structure of PHFTG. Therefore, one‐step separation process (only 30 kDa membrane) was designed to produce fuco‐xylo‐galacturonan from depolymerized TG. This method could simplify the method of the previous study (Gavlighi, Michalak, et al., 2013), which used two‐step membrane separation with 2 and 10 kDa membranes to produce prebiotic fractions of TG.

**TABLE 1:** Sugar composition of pectinase hydrolyzed fraction of tragacanth gum with molecular weight >30 kDa (mg/g dry matter)

Sugar	Concentration (mg/g dry matter)
Fucose	217.00 ± 20
Rhamnose	21.00 ± 10
Arabinose	86.00 ± 40
Galactose	19.60 ± 0.90
Glucose	0.89 ± 0.43
Xylose	296.00 ± 20
Galacturonic acid	351.00 ± 30

Note

Data are mean ± *SD* (*n* = 3).

The data obtained from HPSEC‐UV‐MALLS‐RI showed that the *M*_w_ of PHFTG was 147.7 g/mol, while the corresponding value of intact TG, determined by using Svedberg's method, has been reported to be about 840 kDa (Gralén & Kärrholm, 1950). The specific volume of gyration (SV_g_) is measured to give the theoretical gyration volume (cm^3^) per unit of molar mass (g), providing the mass‐based information on the density. Additionally, the SV_g_ is inversely correlated with the degree of molecular compactness of polysaccharide (You & Lim, 2000). SV_g_ of PHFTG was 2.73 cm^3^/g, whereas Raoufi, Kadkhodaee, Fang, and Phillips (2019) reported the SV_g_ value of 6.93 cm^3^/g for intact TG (*A. gossypinus*). It suggested that the polysaccharide molecules of TG had a less compact and looser structure with expanded conformation as compared to PHFTG. The radius of gyration (*R*_g_) is one of the most commonly used parameters to estimate the approximate size of molecules. In the current study, the size of the PHFTG was roughly estimated by the calculated *R*_g_, which showed a value of 54.1 nm. There are no data on *R*_g_ of intact TG, but Mohammadifar, Musavi, Kiumarsi, and Williams (2006) reported the *R*_g_ of 95.4 nm for tragacanthin fraction of TG (*A. gossypinus*). Results of *M*_w_ and SV_g_ confirmed that long‐chain TG and highly branched structure of TG were hydrolyzed during enzyme treatment.

### 3.2 Prebiotic activity

Growth characteristic of the *L. casei* in the presence of inulin, TG, and PHFTG was determined by measuring pH and acidity of media and the bacterial population in 24‐hr intervals for 48 hr at 37°C, with glucose or inulin as the positive controls and GF‐MRSB as a negative control. As seen in Table 2, glucose induced the highest growth stimulation and lactic acid production when compared to other carbon sources during the whole fermentation period. This could be associated with the high availability of simple carbon sources for *L. casei* utilization. As explained by Huebner, Wehling, Parkhurst, and Hutkins (2008), the prebiotic potential of a substrate alludes to its ability to assist the growth of probiotic bacteria in relation to growth on a nonprebiotic substrate such as glucose.

**TABLE 2:** Changes in pH, total titratable acidity (%), and populations of *Lactobacillus casei* (log CFU/ml) in glucose‐free MRSB medium contain TG, PHFTG, and inulin at inoculation level of 4 log CFU/ml

Prebiotic type	Concentration (%)	24 hr			48 hr		
		Microbial population	pH	Acidity	Microbial population	pH	Acidity
Control	0	6.51 ± 0.01^g^	6.29 ± 0.01^a^	0.18 ± 0.00^e^	6.57 ± 0.01^f^	6.26 ± 0.05^a^	0.17 ± 0.00^f^
TG	0.5	7.69 ± 0.00^d^	6.04 ± 0.02^b^	0.24 ± 0.01^d^	7.64 ± 0.00^e^	6.10 ± 0.04^ab^	0.24 ± 0.01^e^
	1	7.80 ± 0.00^b^	6.01 ± 0.00^bc^	0.29 ± 0.01^c^	7.85 ± 0.06^d^	6.07 ± 0.07^ab^	0.26 ± 0.00^e^
PHFTG	0.5	7.71 ± 0.01^cd^	5.90 ± 0.02^c^	0.45 ± 0.00^b^	8.88 ± 0.00^a^	5.54 ± 0.08^c^	0.61 ± 0.00^c^
	1	7.77 ± 0.01^bc^	5.69 ± 0.00^d^	0.49 ± 0.01^b^	8.94 ± 0.01^a^	5.38 ± 0.03^c^	0.70 ± 0.00^b^
Inulin	0.5	7.12 ± 0.00^f^	6.15 ± 0.01^ab^	0.24 ± 0.00^d^	8.71 ± 0.00^bc^	6.02 ± 0.01^ab^	0.29 ± 0.01^e^
	1	7.20 ± 0.00^e^	6.13 ± 0.02^ab^	0.26 ± 0.01^cd^	8.82 ± 0.01^ab^	5.88 ± 0.02^b^	0.36 ± 0.01^d^
Glucose	2	8.11 ± 0.00^a^	4.14 ± 0.06^e^	1.40 ± 0.01^a^	8.60 ± 0.00^c^	4.05 ± 0.03^d^	1.84 ± 0.02^a^

Note

For each parameter, values (average ± *SD*) in the same column with the same lowercase letter are not significantly different (*p* > .05).

Abbreviations: PHFTG, pectinase hydrolyzed fraction of tragacanth gum; TG, tragacanth gum.

The number of bacterial cells incubated with both concentrations of TG, PHFTG, and inulin as sole carbon source was significantly higher than that of negative control after 24‐ and 48‐hr fermentation (*p* < .05), indicating *L. casei* could utilize all investigated carbohydrate sources (Table 2). During the first 24‐hr fermentation, TG and PHFTG stimulated the proliferation of *L. casei* to a greater extent compared with inulin, known as commercial prebiotic. PHFTG and inulin had a stimulatory effect on cell growth until the end of incubation, whereas the viability of *L. casei* in TG‐supplemented media was approximately stable during the second 24 hr. This result demonstrates that *L. casei* showed selectivity for assimilation of individual prebiotics during second day of incubation, which followed the order: PHFTG 1% > PHFTG 0.5% > inulin 1% > inulin 0.5% > glucose 2% > TG 1% > TG 0.5 (Table 2). Various polysaccharides or oligosaccharides exhibited different prebiotic activities depending on their structural properties including sugar composition, type of linkages, molecular weight (Gavlighi, Michalak, et al., 2013), solubility and viscosity (Wang et al., 2015), and fermentation pathways and uptake mechanisms of probiotic strains (Li et al., 2020). The putative fucosidase activity of some probiotic strains was expected as a possible way in the utilization of linked *L*‐fucose residues present in plant‐derived fucosyl‐oligosaccharides or human milk oligosaccharides. It was reported that the genome of *B. longum* subsp. *infantis* (Gavlighi, Michalak, et al., 2013) and *L. casei* BL23 (Rodríguez‐Díaz, Monedero, & Yebra, 2011) carries fucosidase‐encoding genes. From the sugar composition point of view, TG or PHFTG could display prebiotic activity mainly because of the *L*‐fucose‐substituted saccharide, which also recognized as being responsible for bifidogenic potential of some human milk oligosaccharide structures (as α‐l → 2, α‐l → 3, and α‐l → 4 substitutions; Mikkelsen et al., 2014). However, the higher molecular weight (840 kDa), complex structure, and high viscosity‐inducing effects of TG presumably prevent unfolding of this potential as compared to PHFTG with a molecular weight of 147.7 kDa. This result coincided with the report that probiotic strains preferred to utilize partially hydrolyzed guar gum than native gum due to lack of essential enzymes that assist in breaking down of the branched structure of the later (Mudgil, Barak, Patel, & Shah, 2018). Gavlighi, Michalak, et al. (2013) found that growth promotion of three *B. longum* subsp. *infantis* strains on low molecular weight fractions of TG (*M*_w_ < 2 kDa and 2 < *M*_w_ < 10) was better than commercial galactan and high molecular weight fraction (>10 kDa). Moreover, low viscosity and high solubility are other substantial factors that enhance the accessibility of polysaccharides prebiotic to probiotic. It should be mentioned that no perceptible changes have been seen in appearance and viscosity of GF‐MRSB media after the addition of PHFTG and inulin in comparison with the markedly increased viscosity due to TG incorporation. As reported by Huang et al. (2019), a similar result was observed for a polysaccharide extracted from longan pulp with superfine grinding‐assisted enzymatic technique. This resulted in a significant increase in the proliferation of *L. plantarum*, *L. bulgaricus*, *L. fermentum*, and *L. mesenteroides* due to its low viscosity and high solubility as compared to the use of hot water and superfine grinding extracts.

The metabolism of TG, PHFTG, and inulin was accompanied by a progressive decline in the pH of GF‐MRSB, which implied that *L. casei* was able to utilize them. As seen in Table 2, TTA increment was concurrent with the order of the *L. casei* population when assimilated glucose, TG, PHFTG, and inulin. Also, pH and TTA correlate with the concentration of prebiotics during the whole fermentation period. The most considerable pH decline and TTA increase were seen in glucose‐supplemented GF‐MRSB, which was associated with the high availability of simple sugars for *L. casei* usage. Probiotics hydrolyze polysaccharide substrate into monosaccharides, and they subsequently converted them into short‐chain fatty acids, including acetate, propionate, butyrate, and lactate (Akbari‐Alavijeh, Soleimanian‐Zad, Sheikh‐Zeinoddin, & Hashmi, 2018). This phenomenon finally leads to a considerable increase in TTA of the fermented medium. The decreases in media pH with TG at both concentrations (6.07–6.10) were almost the same as those with inulin (5.88–6.02; *p* > .05). However, the bacterial metabolism of PHFTG causes a more significant decline in the pH of GF‐MRSB (5.38–5.54).

### 3.3 pH and TTA of yogurts

**FIGURE 2:**
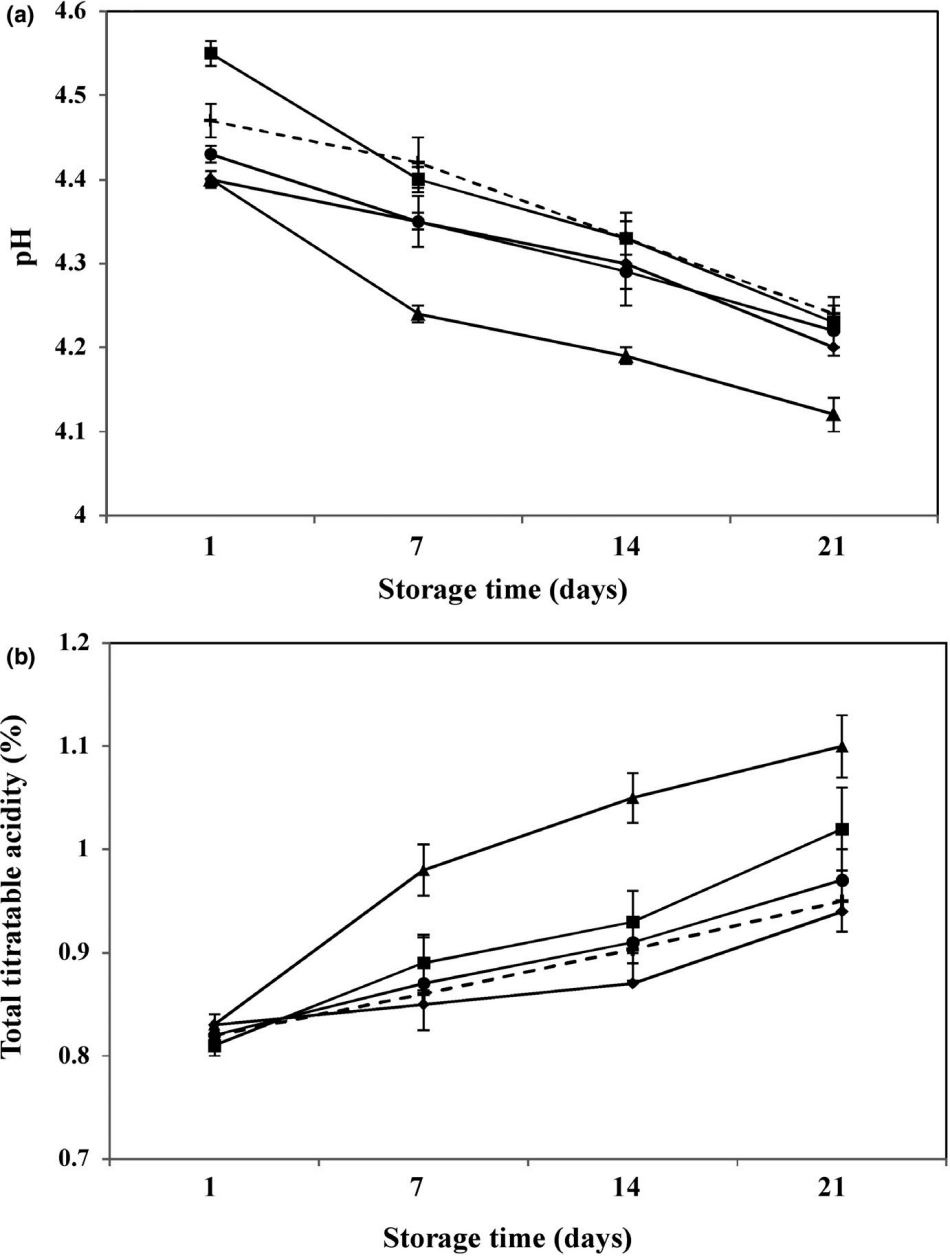
pH (a) and total titratable acidity (as lactic acid%) (b) of different formulations of low‐fat yogurt during storage at 4°C. Control (+): yogurt without both *Lactobacillus casei* and prebiotic; LC‐Cont (♦): yogurt containing *L. casei*; LC‐In (■): yogurt containing *L. casei* and 0.5% inulin; LC‐PHFTG (▲): yogurt containing *L. casei* and 0.5% pectinase hydrolyzed fraction of tragacanth gum; LC‐TG (●): yogurt containing *L. casei* and 0.05% tragacanth gum. Error bars represent standard deviation (*n* = 3)

Acidity and pH are the main attributes for assessment of yogurt quality, and pH reduction plays a crucial role in the formation of the three‐dimensional structure and semisolid texture during fermentation (Sah et al., 2016). Our results revealed that the prebiotic type and storage time had a profound effect on the pH value of the formulated yogurt (*p* < .05). After one day of cold storage, pH varied from 4.4 to 4.55 among all yogurts (Figure 2a), and in contrast to other synbiotic yogurts, LC‐In yogurt had higher pH values compared with Control (*p* < .05). Similar results were reported by Guven Yasar Karaca and Hayaloglu (2005) who observed a slight increase in low‐fat yogurt pH after fortification with 1% and 3% inulin. The postacidification effect was observed in all yogurts (Figure 2a), which is primarily related to the continuity of lactose fermentation by starter culture bacteria during the storage period (Basiri, Haidary, Shekarforoush, & Niakousari, 2018). This can be seen by the small decrease of pH in Control and LC‐Cont samples without any prebiotics. At the end of the storage, LC‐In and LC‐PHFTG yogurts displayed a pH drop of ~0.32 and 0.28 units, while those of the Control, LC‐Cont, and LC‐TG only declined ~0.23, 0.20, and 0.21 units as compared to the first day, respectively. These results are in agreement with Demirci, Aktaş, Sözeri, Öztürk, and Akın (2017) who reported that pH values of *L. casei* 431 inoculated yogurts produced with or without rice bran were fairly similar without noticeable difference between them. It is assumed that, in the presence of PHFTG, *L. casei* is potentially more active than TG, as confirmed by high lactic acid production and consequently decreasing pH in yogurt. Similarly, Aryana and McGrew (2007) proclaimed that yogurt fortified with short‐chain inulin had a lower pH than those contained medium and long‐chain inulin, which is explained by faster consumption of the former by *L. casei*.

Lactic acid is the most prevalent acid produced by probiotic bacteria (Gunenc et al., 2016). In this study, the TTA values distinctly depended on the type of prebiotic,* L. casei*, in the presence of PHFTG and inulin, giving a higher concentration of lactic acid as compared to TG and controls (Figure 2b). As the storage time is extended, the starter culture of yogurt ferment lactose, in turn, increases the TTA of all samples. As mentioned in Section 3.2, *L. casei* could utilize available carbohydrate sources such as PHFTG and inulin faster than TG, which resulted in significantly higher TTA of LC‐PHFTG and LC‐In on day 21 as compared to the first day (*p* > .05). The identical results were evident for a study on the effect of hot and cold break tomato powders on the quality of set‐type yogurt contained *L. paracasei* subsp. *paracasei* F19 (Demirci et al., 2020).

### 3.4 Syneresis and WHC of yogurts

Textural attributes of some dairy products have been reported to be changed dramatically at the high concentration of TG (Aziznia et al., 2008; Cooke et al., 2013). In this context, the choice of a suitable concentration is crucial to avoid a textural defect of yogurt during manufacturing and storage. Results of our preliminary trials showed that semisolid texture of yogurt did not form after incubation at both concentrations of TG, which are used for prebiotic activity experiments. Hence, different concentrations of TG (0.25%, 0.1%, 0.075%, and 0.05%) have been tried, and finally, TG at 0.05% concentration was selected to produce yogurt without severe syneresis or textural defect (data were not shown). Our findings were similar to Aziznia et al. (2008), who reported greater syneresis of nonfat yogurt upon TG addition at above 0.5 g/L concentration. Indeed, TG carries carboxylic groups and considers as an anionic polysaccharide. By pH reduction from 4.43 to 4.22 during acidification (Figure 2a), the negatively charged TG molecules may interact electrostatically with the positive groups on the surface of casein micelles. The conformational changes in adsorbed TG could increase electrostatic repulsion of casein micelles and contribute to a higher whey separation from a weaker and more open structure of TG‐enriched yogurt (Aziznia et al., 2008).

**FIGURE 3:**
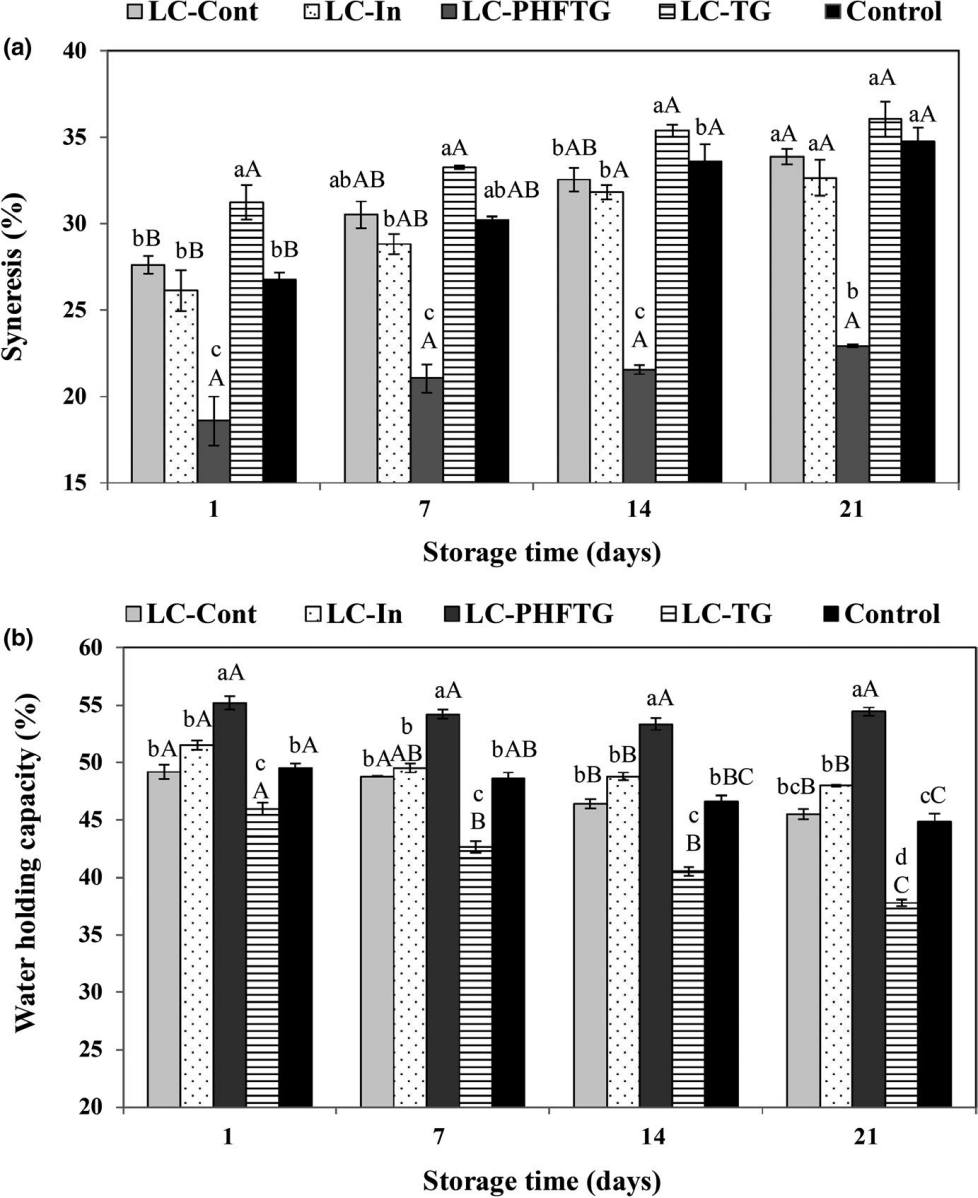
Syneresis (%) (a) and water‐holding capacity (%) (b) of different formulations of low‐fat yogurt during storage at 4°C. Control: yogurt without both *Lactobacillus casei* and prebiotic; LC‐Cont: yogurt containing *L. casei*; LC‐In: yogurt containing *L. casei* and 0.5% inulin; LC‐PHFTG: yogurt containing *L. casei* and 0.5% pectinase hydrolyzed fraction of tragacanth gum; and LC‐TG: yogurt containing *L. casei* and 0.05% tragacanth gum. Different lowercase letters show the significant differences (*p* < .05) between the samples in the same storage time, and different uppercase letters indicate significant differences (*p* < .05) between the storage times of each yogurt samples. The error bars represent the standard deviation (*n* = 3)

Coagulum stability is one of the main quality parameters of set‐type yogurts, which should be monitored during storage (Srisuvor et al., 2013). Spontaneous syneresis is defined as the expulsion of whey from the body of yogurt that arises from the weakening of the gel network (Lucey & Singh, 1997). All yogurt samples presented a varying degree of whey separation with the values of 18.58%–31.23% at the beginning of the experiment (*p* > .05). As depicted in Figure 3a, the longer the storage time of the yogurts, the more the syneresis percentage for all yogurt samples. On day 21, syneresis percentage values increased in the samples in the following order: LC‐TG (36.04) > Control (34.76) > LC‐Cont (33.88) > LC‐In (32.66) > LC‐PHFTG (22.94). Generally, LC‐PHFTG yogurt retained a significantly greater percentage of whey within its structure (*p* < .05), thus being characterized by the lowest syneresis among all samples during the whole storage time. On the contrary, TG‐incorporated yogurts displayed visually whey separation even from day 1. LC‐TG yogurt has greater syneresis values compared with the Control and LC‐Cont yogurt throughout the storage period, reflecting the weakness of gel possibly due to the thermodynamic incompatibility of TG with milk proteins (Tolstoguzov, 2003). These results are in agreement with previous studies (Vasiljevic, Kealy, & Mishra, 2007) and may be explained by the presence of long‐chain polysaccharides, which may interfere with the creation of a three‐dimensional network of casein, leading to a weaker gel with increased wheying off. Similarly, higher whey separation in fiber‐rich pineapple peel powder‐fortified probiotic yogurt was reported owing to flocculation reduction of the casein micelles in the presence of these polymers (Sah et al., 2016). Syneresis was slightly lower for LC‐In than for Control and LC‐Cont (*p* > .05). The lower susceptibility of inulin‐containing yogurt to syneresis was previously reported for reduced‐fat stirred yogurt, and it was attributed to the interaction of hydroxyl groups of inulin with the charged group on the surface of the milk protein (Crispín‐Isidro, Lobato‐Calleros, Espinosa‐Andrews, Alvarez‐Ramirez, & Vernon‐Carter, 2015).

Water‐holding capacity is though a desirable attribute of yogurt and associated with the ability of the proteins and polysaccharides to retain water in the yogurt gel structure. Fortification with prebiotics had a remarkable influence on the WHC, representing the values of 45.98% to 55.19%, respectively, for the first day (Figure 3b). Both inulin and PHFTG appear to improve the tendency of the yogurts to retain water in comparison with Control and LC‐Cont samples. With the exception of PHFTG, which brings the nearly constant WHC during the entire storage period (53.33%–55.19%; *p* > .05), the percent of water retention was statistically decreased for other formulations with extended storage time (*p* < .05). This contrasted with the findings of Demirci et al. (2017) where rice bran was found to limit syneresis of yogurt, possibly reflecting it has dietary fibers such as β‐glucan, pectin, galactooligosaccharide, hemicellulose, and arabinogalactan, which have considerable water‐binding ability. As can be seen in Figure 3a,b, the LC‐PHFTG yogurt had higher WHC and resistance toward phase separation as compared to other yogurts. This may be explained by the increased number of newly absorption sites (chain end group) per unit weight of PHFTG through enzyme depolymerization of initial TG and, consequently, greater water absorption (Wang et al., 2013). Accordingly, lower syneresis percentage of LC‐PHFTG may be explained by the fact that with decreasing the molecular weight of PHFTG, its interference with casein micelles during the creation of the gel network decreases in comparison with long chains of native TG.

### 3.5 Viability of *L. casei* during yogurt storage

**FIGURE 4:**
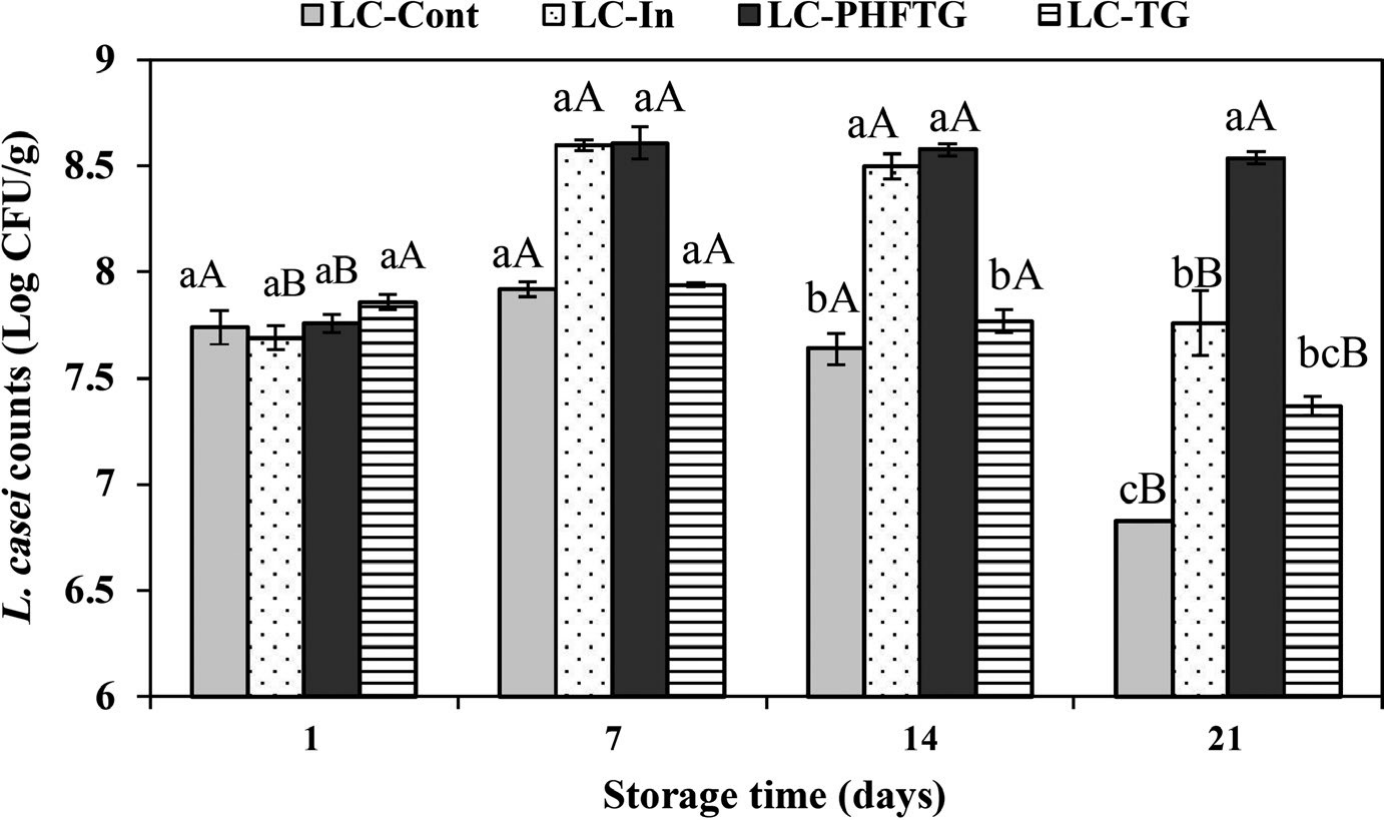
Viability of *Lactobacillus casei* in different formulations of low‐fat yogurt during storage at 4°C. Control: yogurt without both *L. casei* and prebiotic; LC‐Cont: yogurt containing *L. casei*; LC‐In: yogurt containing *L. casei* and 0.5% inulin; LC‐PHFTG: yogurt containing *L. casei* and 0.5% pectinase hydrolyzed fraction of tragacanth gum; and LC‐TG: yogurt containing *L. casei* and 0.05% tragacanth gum. Different lowercase letters show the significant differences (*p* < .05) between the samples in the same storage time, and different uppercase letters indicate significant differences (*p* < .05) between the storage times of each yogurt samples. The error bars represent the standard deviation (*n* = 3)

Despite the health benefits proposed for the supplementation of dairy products with probiotic bacteria, the primary challenge is to maintain the bacterial survival rate above the critical threshold throughout storage at refrigeration temperature (Fazilah, Ariff, Khayat, Rios‐Solis, & Halim, 2018). Functional efficacy and viability of probiotics in yogurts are complex phenomena and depend on such varied parameters including the species/strains used, coculture and species interaction, inoculation practice, product acidity, amount of dissolved oxygen, storage time and temperature, growth promoters and inhibitors, the chemical composition of the carrier medium, and availability of nutrients (Lourens‐Hattingh & Viljoen, 2001). Incorporation of prebiotic substances is one of the most effective approaches that have been conducted to retain the viability of probiotic during shelf life of yogurts. Altogether, the ANOVA revealed that yogurt formulation, storage time, and their interaction significantly influenced *L. casei* counts during yogurt storage (*p* < .05). In accordance with the results of prebiotic activity in broth medium, *L. casei* presented a distinct growth pattern in the presence of different prebiotics during cold storage of yogurt samples. As shown in Figure 4, *L. casei* counts in all yogurt samples displayed a steady increase up to 7 days of storage. The highest *L. casei* counts were observed on day 7 for all samples. Surprisingly, the counts of *L. casei* were always higher than 8.5 log CFU/g in LC‐PHFTG sample from day 7, and after that, the counts seemed to be stagnant. The *L. casei* count of LC‐In yogurts appeared significantly lower than LC‐ PHFTG on day 21 (*p* < .05; Figure 4). As opposed to LC‐PHFTG, the results showed that LC‐TG and mainly LC‐Cont yogurts provided conditions that led to a remarkable decrease in survivability, especially after day 7. Regardless of the report that accumulation of organic acids and yogurt pH below 4.3 had an adverse effect on the viability of probiotics (Lankaputhra, Shah, & Britz, 1996), however, our results disproved this statement since the increase in TTA values (Figure 3b) had no noticeable impact on *L. casei* viability. *L. casei* count in LC‐PHFTG, LC‐In, and LC‐TG was 1.71, 0.93, and 0.53 log CFU/g higher than LC‐Cont yogurt at the end of cold storage. There are no studies on the prebiotic potential of native or hydrolyzed TG in yogurts and other dairy products. *L. paracasei* displayed notable viability in yogurts supplemented with Zedo gum and spirulina, which attributed to the consumption of the hydrolyzed Zedo gum by probiotic during the final days of storage (Alizadeh Khaledabad, Ghasempour, Moghaddas Kia, Rezazad Bari, & Zarrin, 2020). In accordance with our findings, Demirci et al. (2017) reported that counts of *L. casei* 431 were above 8 CFU/g in yogurt fortified with 2%–3% rice bran after 21 days of cold storage. Bosnea, Kopsahelis, Kokkali, Terpou, and Kanellaki (2017) stated that *L. casei* is considered as an acid‐tolerant LAB, which counts remained above 10^7^ CFU/g after 60 days of cold storage in yogurt supplemented with freeze‐dried wheat. Also, in a previous study, Costa et al. (2019) pointed out that oligofructose showed pronounced protection on the viability of *L. casei* as compared to natural sugar substitutes (stevia, erythritol, or xylitol) or polydextrose during 4 weeks shelf life of yogurt. Aqueous extract of *Auricularia auricula* was found to be effective on the growth of *Lactobacillus acidophilus La‐5* and *Bifidobacterium bifidum* Bb‐12 strains in synbiotic yogurt during cold storage, which was attributed to prebiotic stimulation potential of its polysaccharide compounds (Faraki, Noori, Gandomi, Banuree, & Rahmani, 2020).

Our current findings show that supplementation of yogurt with inulin, TG, and especially PHFTG as growth promoters could be an acceptable procedure of maintaining the count of *L. casei* above the minimum recommended level of 10^7^ log CFU/g to certify their health benefits (Stanton et al., 2005). By considering the highest prebiotic activity of PHFTG in our research, it remains unclear that PHFTG improves *L. casei* survival by providing additional nutrients after digestion by extracellular enzyme (Makras, Acker, & Vuyst, 2005) or by modification of negative environmental parameters and protecting cells from injury (Vasiljevic et al., 2007).

### 3.6 Sensory acceptance of yogurt

High‐quality yogurt should possess satisfying aroma and taste and, at the same time, retain its curd stability without any symptom of shrinkage, lumps, and whey separation (Srisuvor et al., 2013). The scores collected for appearance, flavor, mouthfeel, body and texture, syneresis, and overall acceptability of different yogurt formulations are displayed in Table 3. The samples that presented mean scores higher than 3 on a scale of 1–5 were considered acceptable. Except for flavor, the three investigated prebiotics change preference of various attributes of the yogurt in comparison with the Control and LC‐Cont (Table 3). Incorporation of *L. casei* has been shown to not significantly change all of the investigated sensory properties of LC‐Cont when compared to Control (*p* > .05). These findings are in agreement with Hekmat and Reid (2006), who reported sensory characteristics of 1% fat yogurt did not change significantly after incorporation of *L. reuteri* RC‐14 and *L. rhamnosus* GR‐1, although Cruz et al. (2010) declared that probiotic metabolism may negatively affect taste and odor of the final probiotic product due to the production of some components.

**TABLE 3:** Sensory scores of low‐fat yogurts with different formulations

Yogurt formulation	Sensory attributes					
	Appearance	Flavor	Mouthfeel	Body and texture	Visual syneresis	Overall acceptability
Control	4.24 ± 0.94^a^	4.16 ± 0.73^a^	3.88 ± 1.24^bc^	3.76 ± 1.10^b^	3.16 ± 1.31^b^	3.44 ± 0.69^b^
LC‐Cont	4.28 ± 1.28^a^	4.40 ± 0.80^a^	3.92 ± 1.09^bc^	3.84 ± 0.96^ab^	3.32 ± 0.67^b^	3.44 ± 0.85^b^
LC‐In	4.52 ± 0.98^a^	4.32 ± 0.78^a^	4.56 ± 0.63^ab^	4.32 ± 0.78^ab^	3.92 ± 1.09^ab^	4.40 ± 0.63^a^
LC‐PHFTG	4.64 ± 0.68^a^	4.24 ± 0.70^a^	4.76 ± 0.43^a^	4.56 ± 0.63^a^	4.28 ± 0.72^a^	4.36 ± 0.74^a^
LC‐TG	3.40 ± 1.05^b^	4.00 ± 0.69^a^	3.64 ± 1.05^c^	2.60 ± 1.05^c^	2.12 ± 0.90^c^	2.72 ± 0.77^c^

Note

For each attributes, values (average ± *SD*) in the same column with the same lowercase letter are not significantly different (*p* > .05).

Abbreviations: Control: yogurt without both *Lactobacillus casei* and prebiotic; LC‐Cont, yogurt containing *L. casei*;LC‐In, yogurt containing *L. casei* and 0.5% inulin; LC‐PHFTG, yogurt containing *L. casei* and 0.5% pectinase hydrolyzed fraction of tragacanth gum; LC‐TG, yogurt containing *L. casei* and 0.05% tragacanth gum.

Assessors did not perceive any significant differences among different yogurt formulations in terms of flavor (*p* > .05), and flavor scores of all the yogurts were found to be above the acceptable values. Similarly, Srisuvor et al. (2013) did not find flavor and odor differences among low‐fat set yogurts added with 1–3 g/100 g of inulin or polydextrose as prebiotic in comparison with control.

Table 3 pinpointed that LC‐PHFTG, LC‐In, LC‐Cont, and Control were perceived as having significantly more desirable visual attributes than LC‐TG (*p* < .05). The lower appearance ranking for the LC‐TG yogurt seemed to be associated with its higher syneresis, the existence of particles, and the small clots in the separated whey phase and visible cracks on the surface. Demirci et al. (2017) reported that appearance and color ratings of yogurts were significantly decreased by incorporation of 1%–3% rice bran. Likewise, appearance sensory attributes, including color, aspects of surface, and glossiness of low‐fat yogurts, were not significantly affected by supplementation with inulin and partially hydrolyzed guar gum (Brennan & Tudorica, 2008).

In our study, assessors judged the texture of yogurts by both mouthfeel and visual examination by spoon. As can be concluded from Table 3, yogurt supplemented with TG received the lowest body and texture score (2.60), below the acceptable value, as perceived by spooning. Incorporation of TG led to the coarser and much more open structure with visible lumps than other yogurts, possibly due to interference with milk protein during curd formation and the creation of the large pores in yogurt body. Indeed, the addition of TG deteriorates the coherent and uniform structure of yogurt. Similarly, Hasani, Sari, Heshmati, and Karami (2017) stated that nonmouthfeel texture score of low‐fat yogurt decreased by incorporation of barely bran as prebiotic. LC‐PHFTG yogurt showed significantly smoother and more compact and fine textures with higher texture acceptance (4.56) than both controls and LC‐In yogurts.

PHFTG provides suitable mouthfeel to the yogurt in which it is added, and the assessors expressed LC‐PHFTG, and LC‐In had a more sensible creamy mouthfeel. The lowest mouthfeel ranking (3.64) was received by LC‐TG yogurt, followed by Control (3.88) and LC‐Cont (3.99). Most of the assessors stated the coarse texture was easily felt in LC‐TG. In line with our findings, it was reported that the mouthfeel score of stirred yogurt was significantly decreased after addition of carboxy methyl cellulose, flaxseed mucilage, or their combination (Basiri et al., 2018).

It was evident that PHFTG could successfully retard whey‐off from the product until day 10, and the results of visual evaluation of syneresis are compatible with those observed instrumentally in Figure 3a. The syneresis score of LC‐TG (2.12) was significantly below the rejection limit (*p* < .05), whereas syneresis of LC‐In, LC‐Cont, and Control was not judged as unacceptable by assessors.

Overall acceptability is a complex and multidimensional concept that consists of different sensory attributes including flavor, texture, and appearance perceptions. Panelists evaluated the overall acceptability of yogurt formulations with scores between 2.72 and 4.40, indicating that they disliked slightly LC‐Cont and Control and liked LC‐PHFTG and LC‐In formulations, noticeably. From the standpoint of overall preference, it could be observed that mouthfeel, texture, and appearance of LC‐TG yogurt were deteriorated by adding the 0.05% TG as prebiotic substance, providing it with inferior overall preference below the rejection limit (2.72; Table 3). Mudgil et al. (2016) found that partially hydrolyzed guar gum could be enhancing the overall acceptability of yogurt at 2.5%–3% level. Yogurts are multidimensional food products with respect to organoleptic characteristics. Incorporation of probiotic and prebiotic increases this complexity due to additional metabolic activities of cultures. Hence, it is crucial to evaluate consumer perception with sensorial methodologies including performance of check‐all‐that‐apply, projective mapping, sorting, and intensity scales in future studies and during development of new products (Cruz et al., 2013).

## 4 CONCLUSION

In our study, the combination of the enzyme depolymerization and membrane separation with one‐step process was employed to generate a novel hydrolyzed fraction from TG with fucoxylogalacturonan structure and prebiotic potential. It was identified that low molecular weight TG was easily fermented as indicated by concomitant increases in *L. casei* population and decrease in media pH when compared to inulin or native TG. PHFTG can be incorporated into yogurt successfully at a level of 0.5%, and this yogurt kept its optimum properties even up to 3 weeks in refrigerated storage. On the other hand, the use of TG (0.05%) resulted in deterioration of yogurt texture, higher syneresis, and low sensorial score, so it could not be utilized as a prebiotic or fat replacer in low‐fat yogurt to simulate high‐fat product. We believe that hydrolyzed gums can be suitable substitutes for conventional stabilizers or fat replacer in the food industry that can also enhance the health benefits of products.

## CONFLICT OF INTEREST

The authors declare that there are no conflicts of interest.

## ETHICAL STATEMENT

This study does not involve any human or animal testing.
